# Is necrotizing enterocolitis associated with the 896A/G polymorphism in the toll-like receptor 4 (TLR4) gene?

**DOI:** 10.1590/0102-672020260000030e1959

**Published:** 2026-08-31

**Authors:** Danielle Lopes Teixeira FERDINANDO, Alexandre Alberto Barros DUARTE, Vânia Belintani PIATTO, Heloisa Cristina CALDAS

**Affiliations:** 1 Faculdade de Medicina de São José do Rio Preto, Pediatrics and Pediatric Surgery – São José do Rio Preto (SP), Brazil.; 2 Faculdade de Medicina de São José do Rio Preto, Anatomy Unit – São José do Rio Preto (SP), Brazil.; 3 Faculdade de Medicina de São José do Rio Preto, Molecular Biology – São José do Rio Preto (SP), Brazil.

**Keywords:** Enterocolitis, necrotizing, Toll-like receptor 4, Polymorphism, genetic, Enterocolite necrosante, Receptor 4 toll-like, Polimorfismo genético

## Abstract

**Background:**

The pathophysiology of necrotizing enterocolitis (NEC) involves changes in intestinal development that hinder its functionality, leading to both metabolic and gene and phenotypic changes. Among the genetic factors the 896A/G polymorphism in the *Toll-Like Receptor 4 (TLR4)* gene can trigger is an inappropriate and persistent inflammatory response, leading to the progression of lesions and necrosis of the intestinal mucosa, and reduced perfusion of the microvasculature, increasing susceptibility to the disease.

**Aims:**

To determine the prevalence of the 896A/G polymorphism in the TLR4 gene in neonates with and without NEC.

**Methods:**

Case-control study, in which 100 neonates were evaluated, 50 diagnosed with NEC (Case Group) and 50 without the disease (Control Group), of both sexes. DNA was extracted from peripheral blood leukocytes, and the region encompassing the polymorphism was amplified by polymerase chain reaction/restriction fragment length polymorphism.

**Results:**

Males were predominant in both groups: Cases (54%) and Controls (56%) (p=1.0000). Moderately and extremely preterm infants were the most frequent in the Case (90%) and Controls (96%) (p=0.6132) groups. Very low birth weight and extremely low birth weight neonates were predominant in the Case Group (60%) and in the Control Group (72%) (p=0.0995). Of the 50 neonates with NEC, 66% responded positively to clinical treatment, and 86% were discharged from hospital. The 896A/G polymorphism in the TLR4 gene was not identified in the 200 alleles analyzed (100%).

**Conclusions:**

The absence of the 896A/G polymorphism in the TLR4 gene in NBs with and without NEC does not exclude the possibility of alterations in this and/or other genes, highlighting the importance of additional studies to elucidate this relationship.

## ARTICLE HIGHLIGHTS

Necrotizing enterocolitis (NEC) is a multifactorial disease influenced by intestinal immaturity, inflammation, and genetic susceptibility.

This case-control study evaluated the prevalence of the TLR4 896A/G polymorphism in 100 neonates with and without NEC.

The TLR4 896A/G polymorphism was not identified in any of the 200 alleles analyzed.

These findings suggest that other genetic variants may contribute to NEC susceptibility and warrant further investigation.

## Summary

**CENTRAL MESSAGE:**

Necrotizing enterocolitis (NEC) is a multifactorial disease associated with risk factors such as intestinal immaturity, dysbiosis, hypoxia, low gestational age, low birth weight, and genetic predisposition. Its pathogenesis involves complex interactions within the innate immune system, particularly through Toll-like receptor 4 (TLR4), a key mediator of intestinal inflammatory signaling. The TLR4 896A/G polymorphism may alter receptor signaling, promoting dysregulated inflammation, mucosal barrier injury, impaired microvascular perfusion, and intestinal necrosis, thereby increasing susceptibility to NEC and disease severity. However, its absence in this study sample does not exclude the contribution of other genetic variants to NEC pathogenesis.

**PERSPECTIVES:**

Although the present molecular analysis did not identify the TLR4 896A/G polymorphism in NBs with or without NEC, these findings should be interpreted cautiously. NEC is a multifactorial disease involving complex genetic interactions, and the absence of this polymorphism does not exclude the contribution of other variants in TLR4 or related genes. Clarifying the role of TLR4 polymorphisms in NEC may provide valuable insights for risk stratification, prevention, and targeted therapeutic strategies, particularly in preterm infants, who are highly vulnerable to intestinal barrier dysfunction and excessive inflammation. Further studies are needed to better define this association.

## VISUAL ABSTRACT

**Figure:**
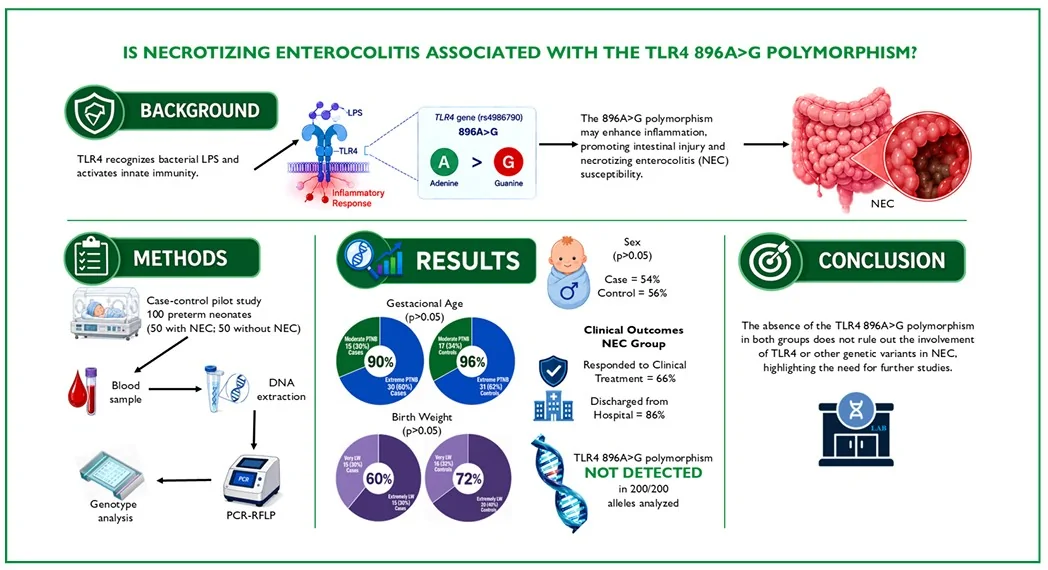

## RESUMO

**Racional:**

A fisiopatologia da enterocolite necrosante (ECN) envolve alterações no desenvolvimento intestinal que comprometem sua funcionalidade, levando a modificações metabólicas, na expressão gênica e fenotípica. Entre os fatores genéticos, o polimorfismo 896A/G, no gene *Toll-Like Receptor 4 (TLR4)*, pode desencadear uma resposta inflamatória inadequada e persistente, levando à progressão da lesão, à necrose da mucosa intestinal e à redução da perfusão da microvasculatura, aumentando a suscetibilidade à doença.

**Objetivos:**

Determinar a prevalência do polimorfismo 896A/G no gene TLR4, em neonatos com e sem ECN.

**Métodos:**

Estudo de caso-controle, no qual foram avaliados 100 neonatos, sendo 50 diagnosticados com ECN (Grupo Casos) e 50 sem a doença (Grupo Controles), de ambos os sexos. O DNA foi extraído dos leucócitos do sangue periférico, e a região que abrange o polimorfismo foi amplificada por reação em cadeia da polimerase/polimorfismo no comprimento de fragmentos de restrição.

**Resultados:**

O sexo masculino foi predominante em ambos os grupos: Casos (54%) e Controles (56%) (p=1,0000). Prematuros moderados e extremos foram os mais frequentes nos grupos Casos (90%) e Controles (96%) (p=0,6132). Neonatos de muito baixo peso e extremo baixo peso foram predominantes no Grupo Casos (60%) e no Grupo Controles (72%) (p=0,1073). Dos 50 neonatos com ECN, 66% responderam positivamente ao tratamento clínico e 86% receberam alta hospitalar. O polimorfismo 896A/G no gene TLR4 não foi identificado nos 200 alelos analisados (100%).

**Conclusões:**

A ausência do polimorfismo 896A/G no gene TLR4 em recém-nascidos com e sem ECN não exclui a possibilidade de alterações neste e/ou em outros genes, ressaltando a importância de estudos adicionais para elucidar essa relação.

## INTRODUCTION

 Although no single risk factor has been definitively associated with the development of necrotizing enterocolitis (NEC), others have been suggested as possible triggers. Among them, the following stand out: neonatal asphyxia, umbilical catheterization, early introduction of enteral feeding, patent ductus arteriosus, congenital heart disease, polycythemia, use of indomethacin and/or methylxanthines, and exposure to bacterial lipopolysaccharides^9,12^. Risk factors may occur at any time before the onset of NEC, including the prenatal period. In addition, uncontrollable factors such as gestational age, birth weight and, more recently, genetic predispositions, have also been implicated in the risk of the disease^15,35 ,43^. 

 The premature fetal intestine has increased expression of Toll-Like Receptor 4 (TLR4) receptors as a consequence of their important role in normal intrauterine intestinal epithelial development. However, after premature birth, exposure of the newborn (NB) to hypoxia/endotoxemia causes overstimulation of the still persistently increased expression of intestinal *TLR4* ^10,36,43^. These are then activated by bacteria in the postnatal period, leading to an imbalance between tissue injury and repair and depletion of goblet cells, due to impaired differentiation of intestinal stem cells, histopathological characteristics of NEC. Therefore, *TLR4* plays a fundamental role both in inflammation of the intestinal mucosa and in its basal conditions^27,29,30,40^. 

 Increased expression of *TLR4* in the fetal intestine is a key component in the preparation of the intestine for extrauterine life, influencing the maturation of the intestinal epithelium and the ability to respond appropriately to microbial stimuli. However, dysregulation of this expression, due to polymorphisms, can have negative consequences, such as predisposition to inflammatory bowel diseases, such as NEC^10,27,29 ,30,36 ,40,43^ . 

 Changes in the sequence of several genes belonging to the *TLR* cascade, including the *TLR4* gene, modulate susceptibility to NEC, such as the functional polymorphism 896A/G in the *TLR4* gene. This substitution of an adenine (A) for a guanine (G) at nucleotide position 896, by affecting the extracellular domain of the TLR4 receptors, causes an alteration in the protein sequence, at codon 299, from the amino acid aspartate (aspartic acid — ASP) to glycine (ASP299Gly) and, consequently, in the protein function, thus modifying the interaction of pathogen-associated molecular patterns (PAMPs)^1,8,10,15 ,24,41 ,44,49^ . 

 This polymorphism hinders the function of these receptors in the endothelium and enterocytes, resulting in a reduced response or hyporesponsiveness to bacterial lipopolysaccharide signaling. Furthermore, the absence of negative feedback contributes to the persistence of the inflammatory process and increased susceptibility to infections by enteropathogens^1 ,8,10,15,19 ,20,24 ,38,41 ,44,47 ,49^. 

 Therefore, alterations in the various genes belonging to the *TLR* cascade, such as the 896A/G polymorphism in the *TLR4* gene, can result in an inadequate and chronic inflammatory response leading to the characteristic pathological sequence of injury and necrosis of the intestinal mucosal barrier, with underdevelopment and reduced perfusion of the microvasculature, contributing to increased susceptibility and severity of NEC^1,8,11,15 ,19,20 ,24,28 ,36,38 ,41,44 ,47,49^ . 

 The aim of this study was to verify the prevalence of the 896A/G polymorphism in the TLR4 gene in NBs with and without NEC. 

## METHODS

 Case-control study, which included 100 premature (PTNB) and full-term (FTNB) NBs with and without a diagnosis NEC admitted to the Neonatal Unit of an institution. 

 Modified Bell staging^22,33^ was used for the diagnosis of NEC and the inclusion of PTNB/FTNB. NBs from both groups who presented congenital malformations of the cardiac, digestive, renal and/or respiratory systems, laboratory confirmation of congenital infection, sepsis and/or meningitis, or whose mothers had a history of syphilis, toxoplasmosis, rubella, cytomegalovirus, herpes/human immunodeficiency virus (STORCH/HIV) group infections or drug addiction during pregnancy, use of opiates or respiratory depressant drugs in the peripartum period were excluded. Those whose parents or guardians did not consent to participate in the research were also excluded. 

 The NBs in both groups were classified, according to gestational age, as FTNBs (38–41 weeks and six days), Borderline PTNBs (36–37 weeks and six days), Moderate PTNBs (31–35 weeks and six days), Extreme PTNBs (22–30 weeks and six days)^7,54^. Regarding birth weight, NBs were classified as Appropriate Weight (AWNB)=2,500 g; Low Weight (LWNB) from 1,500 to <2,500 g; Very Low Weight (VLWNB) from 1,000 to <1,500 g; and Extremely Low Weight (ELWNB) <1,000 g^51^ . 

 The following demographic data were also collected from both groups: type of delivery (natural or surgical), type of birth (single or twin), Apgar score at 1 ^st^ and 5^th^ minutes^6^ . The entire sample was composed of patients of the same skin color and from the same geographic region — State of São Paulo, Brazil. 

### Molecular analysis

 Genomic DNA was extracted from peripheral venous blood leukocytes using the GE Illustra Blood Genomicprep Mini Spin KitT (GE Healthcare UK Limited), following the manufacturer’s protocol. The procedure was conducted at the Education and Research Sector of the institution’s Macroscopy Laboratory. The extracted DNA was stored at 4^°^C for 24 hours before being frozen in a -20^°^C freezer. 

 To detect the 869A/G polymorphism (rs4986790), the genomic DNA fragment, which covers the polymorphism region, in the *TLR4* ^42^ gene was amplified by the polymerase chain reaction (PCR) technique with the PCR reagent kit — OneTaqT Hot Start Quick-LoadT 2X Master Mix with Standard Buffer (New England BiolabT) in a final volume of 25 μL, with previously published primers^49^ and further digested by the restriction fragment length polymorphism (RFLP) technique , with 10 U of NcoI enzyme (New England BiolabsT), at 37^°^C/1 hour, with subsequent inactivation at 80^°^C/20’. 

 In the absence of polymorphism 896A/G, in both alleles (wild homozygote — AA), the PCR product is not digested by the NcoI enzyme, because there is no recognition of the restriction enzyme site, showing only a 507 base pairs (bp) fragment. When polymorphism is present in both alleles (polymorphic homozygous — GG), the restriction site of the NcoI enzyme is recognized and the PCR product is digested into two fragments of 488 and 19 bp (=507 bp). In the heterozygous sample (AG), the polymorphism is present in only one allele, therefore the PCR product is composed of three fragments, one of 507 bp (wild-type allele A) and the other of 488 and 99 bp (polymorphic allele G). 

 Products from each of the PCR/RFLP reactions were added to FlashGelT Loading Dye 5x running head dye and electrophoresed in 2.2% agarose gel cassettes on the FlashGelT DNA System to confirm their success, and all the gels were photo-documented by FlashGelT Camera (Lonza Group Ltd Muenchensteinerstrasse 38 CH-4002 Basel Switzerland). To assess genotyping reliability, 10% of the samples were randomly selected for repeat analysis. Repeated genotyping demonstrated 100% concordance with the original results, and no genotyping failures were observed. 

 To avoid biases in molecular investigation and results, all DNA samples were analyzed without knowledge of the information obtained from each patient in the study. 

### Ethical aspects

 The ethics and research protocol were approved by the Institutional Ethics Committee (São Paulo, Brazil) (# 4.410.345/2020). Before starting any procedure, an Informed Consent Form was obtained from all parents or guardians. 

### Statistical analysis

 The results were previously subjected to descriptive statistics to determine normality. For independent samples with normal distribution, the unpaired t-test was used, while the MannWhitney U test was applied for samples with non-normal distribution. To compare qualitative variables, the χ^2^ or Fisher’s exact tests were used, as appropriate. Pearson’s correlation coefficient was calculated to assess the degree of relationship and direction between the variables gestational age and days of life at diagnosis of NEC. When applicable, the Odds Ratio (OR) was calculated, with a 95% confidence interval (95%CI), to study the OR of the event occurring. The significance level adopted was 5%. The results were presented in percentages (%) and, when appropriate, mean (M) and standard deviation (SD) for variables with normal distribution or in median (Med), accompanied by minimum and maximum values, for variables with non-normal distribution. Statistical tests were performed using GraphPad InStat version 3.00, GraphPad Software Inc, San Diego California USA, www.graphpad.com . 

## RESULTS

 The total sample consisted of 100 NBs, of which 50 (50%) were diagnosed with NEC, making up the Case Group, and 50 (50%) were included in the Control Group. Considering the demographic data of the 50 NBs in the Case Group and the 50 NBs in the Control Group, the male sex was predominant in both groups (respectively, 54 and 56%), without statistical significance (p=1.0000). 

 Regarding the NB’s classification the according to gestational age, in both groups there was a higher frequency of Moderate and Extreme PTNB, totaling 90% in the Case Group and 96% in the Control Group. One FTNB (2%) presented clinical and radiographic signs of NEC and was included in the Case Group. The statistical analysis for this demographic variable was not statistically significant (p=0.6132). 

 According to the weight classification, in the Case Group, Very Low Weight and Extremely Low Weight NBs were the most frequent, totaling 60%, and similarly in the Control Group, with a total of 72%. The difference between both groups did not reveal statistical significance (p=0.0995). 

 Regarding the type of delivery, both groups had a higher frequency of surgical delivery, but without statistical significance (p=1.0000). Both groups had a higher frequency of single births, but with statistical significance (p=0.0091). (Table 1). 

**Table 1:** Frequency distribution of demographic characteristics between case and control groups.

Characteristics		Cases N=50 (%)	Controls N=50 (%)	P-value
Sex				
	Male	27 (54)	28 (56)	1.0000^*^
	Female	23 (46)	22 (44)	
Gestational Age Classification				
	Full-Term NB	01 (02)	0 (0)	0.6132^**^
	Borderline PTNB	04 (08)	02 (04)	
	Moderate PTNB	15 (30)	17 (34)	
	Extreme PTNB	30 (60)	31 (62)	
Birth Weight Classification				
	Appropriate Weight	10 (20)	02 (04)	0.0995^***^
	Low Weight (LW)	10 (20)	12 (24)	
	Very LW	15 (30)	16 (32)	
	Extremely LW	15 (30)	20 (40)	
Type of Delivery				
	Natural	12 (24)	13 (26)	1.0000^*^
	Surgical	38 (76)	37 (74)	
Birth Type				
	Single	44 (88)	32 (64)	0.0091^*^
	Multiple (Twins)	06 (12)	18 (36)	

LW: Low Weight; N: Number; NB: newborn; PTNB: Preterm Newborn; Df: degrees of freedom

* Fisher’s Exact Test

** χ^2^ Test (1.808; df:3)

*** χ^2^ Test (6.262; df=3)

### Clinical characteristics of the case and control groups

 Regarding gestational age, both groups presented the same median value (29 weeks), without statistical significance (p=0.3533). 

 The median birth weight was higher for the Cases (1,267.5 g) compared to the Control Group (1,050 g), with statistical significance (p=0.0076). As for the Apgar score, statistical significance was obtained in both indexes: in the 1 ^st^ minute, the median value was 7 for Cases and 5 for Controls (p=0.0289), and in the 5th minute, the median was, respectively, 9 and 8.5 (p=0.0211) (Table 2). 

**Table 2:** Distribution of clinical characteristics between the Cases and Controls groups.

Characteristics		Cases	Controls	P-value^*^
GA (wk)				
	Minimum	24	23	0.3533
	Median	29	29	
	Maximum	38	36	
Weight (g)				
	Minimum	600	500	0.0076
	Median	1,267.5	1,050	
	Maximum	4,034	3,725	
Apgar 1^st^ minute				
	Minimum	0	0	0.0289
	Median	7	5	
	Maximum	9	9	
Apgar 5^th^ minute				
	Minimum	2	2	0.0211
	Median	9	8.5	
	Maximum	10	10	

GA (wk): Gestational Age (weeks)

* Mann-Whitney U test

### Clinical characteristics of the case group

 According to the modified Bell staging system^22,33^, stages II-A and II-B were the most frequent, accounting for 82% of cases. However, among the 17 patients (34%) who required surgical treatment, stages II-B and III-B were the most frequent, in six (35%) and eight patients (47%), respectively. Clinical treatment was successful in 66% of cases, and hospital discharge was successful in 86% of patients with NEC (Table 3). 

**Table 3:** Frequency distribution of the clinical characteristics of the Cases Group.

Characteristics		Cases Group N=50 (%)
NEC Staging		
	II-A	24 (48)
	II-B	17 (34)
	III-B	09 (18)
Treatment		
	Exclusively Clinic	33 (66)
	Surgical	17 (34)
Outcome		
	Hospital Discharge	43 (86)
	Death	07 (14)

NEC: Necrotizing Enterocolitis.

 The distribution of NEC was concentrated between 31 and 38 weeks of postmenstrual age (PMA) with a peak at 36 weeks PMA, with 18 cases (36%) at the time of NEC diagnosis. 

 Pearson’s correlation analysis was performed to verify the association between gestational age (in weeks) and days of life at the time of NEC diagnosis. There was a significantly negative correlation between both variables, indicating that age at NEC diagnosis is inversely related to gestational age at birth (r=-0.5994; p<0.0001). 

### Diagnostic imaging

 Imaging tests (radiographic and ultrasound) allowed the diagnosis of NEC, on the first day of clinical suspicion, according to the following reports found:

- Distension of intestinal loops: 50 cases (100%);
- Pneumatosis intestinalis: 41 cases (82%);
- Portal venous gas: 9 cases (18%);
- Pneumoperitoneum: 5 cases (10%).

### Molecular results

Table 4 presents the genotypic and allelic results found in the Cases and Controls groups, related to the 896A/G polymorphism in the *TLR4* gene. 

**Table 4:** Genotypic and allelic relationship for the 896A/G polymorphism, in the TLR4 gene, between the Cases and Controls groups.

Gene/ Polymorphism	Genotypes/ Alleles	Cases N=50 (%) Alleles N=100 (%)	Controls N=50 (%) Alleles N=100 (%)	OR (CI95%)	P-value
*TLR4/* 896A/G	AA	50 (100)	50 (100)	NA	-----
	AG*	0 (0)	0 (0)		
	GG*	0 (0)	0 (0)		
	A	100 (100)	100 (100)	NA	-----
G*	0 (0)	0 (0)		

OR: odds ratio; CI: Confidence Interval; NA: Not Analyzed

* Polymorphic G allele in bold.

 Among the 200 alleles analyzed, 100% do not present the polymorphic allele (G) of the 896A/G polymorphism in the *TLR4* gene. Therefore, 100% of the neonates in the sample present the homozygous wild genotype (AA). 

## DISCUSSION

 Understanding of the microbial and molecular mechanisms underlying the pathogenesis of NEC, which is still not well understood, has made significant progress. Advances in genetic research have allowed the identification of promising genes associated with increased or decreased risk of NEC, such as the *TLR4* gene^11,53,57^. 

 To date, only one study has investigated the 896A/G polymorphism in the TLR4 gene in PTNB with and without NEC^49^, as carried out in the present research. The reference study sample consisted of 118 PTNB (41 with NEC [35%] and 77 without NEC [65%]), all with birth weight=1,500 g and mean gestational age of 29 weeks (ranging from 24 to 33 weeks), in addition to 116 healthy FTNBs. In all three groups, there was a predominance of males (56, 49 and 49%, respectively)^49^. 

 The main difference in relation to the present study is in the composition of the sample, since both preterm and FTNBs, with and without NEC, were included, without restriction regarding birth weight. However, both studies are similar in terms of the predominance of males in the groups analyzed. 

 No significant differences were observed between the variables analyzed in the present study, as well as in the reference study^49^. However, as mentioned, there was a higher prevalence of males in the total sample. Evidence suggests that sex may influence outcomes in extremely PTNBs^21^, and that heterozygous loci associated with protection against infections are located on the X chromosome^37^. Since males have only one X chromosome, they have fewer of these loci compared to females, thus becoming more vulnerable to the consequences of infectious and inflammatory processes^21,37^. 

 The incidence of NEC among NBs admitted to neonatal intensive care units ranges from 1 to 8%, with a case fatality rate between 10 and 50%. In PTNBs weighing less than 1500 g, this rate ranges from 4 to 13%. Furthermore, approximately 5 to 10% of cases occur in full-term or nearterm NBs^4,16,58^. 

 In the present study, 50% of the total sample presented NEC, with 60% of cases in PTNBs weighing less than 1,500 g and 2% in FTNBs, differing both from the reference study^49^ and from those in the literature^4,16,58^. These differences may be related to the composition of the case study, inclusion and exclusion criteria, ethnicity, geographic region, methodologies adopted, among other factors. 

 The fatality rate observed in the present study (14%) is in agreement with the literature^4,16,58^. Unlike the reference study, which did not present this rate, despite having included extremely premature infants, with very low birth weight, with patent ductus arteriosus, sepsis, bronchopulmonary dysplasia and intracranial hemorrhage — considerable risk factors for death as an outcome^49^. 

 Similarly, the reference study does not report the gestational age at the time of NEC diagnosis^49^. According to the literature, the peak distribution of age at onset of NEC occurs between 29 and 31 weeks of postmenstrual age (PMA)^10,25,26,28,34,39,40,46,55^. 

 In the present study, the distribution of NEC development was concentrated between 31 and 38 weeks of PMA, with a peak at 36 weeks. These findings suggest the need for further investigations, with a larger sample, to elucidate possible factors related to this later onset of NEC. 

 The incidence and postnatal age of onset of NEC are inversely related to birth weight and gestational age of the newborn, suggesting differences in the pathophysiological aspects of the disease between preterm and FTNBs^4,10,16,25,26,28,34,39,40,46,55,58^. These findings are corroborated in the present study, with statistical significance. 

 Diagnosis and management of NEC should be aligned, based on the evaluation of clinical signs and symptoms, as well as abdominal imaging findings, considering frequency peaks according to postmenstrual or corrected age^4,10,16,25,26,28,34,39,40,46,55,58^. Once NEC is suspected, the newborn should undergo a regular imaging protocol to monitor disease progression and guide clinical management. The frequency of this monitoring varies from six to 24 hours, depending on the severity of the condition, and helps in deciding whether surgical intervention is necessary^2,3,17,31,32,48,52^. This protocol was strictly followed in the management of the cases in the present study. 

 Radiographic signs of NEC include localized and generalized bowel distension, bowel wall thickening, pneumatosis intestinalis, air in the hepatic portal venous system, and pneumoperitoneum. However, even with these predictors, the diagnosis of NEC remains a significant challenge for the medical team^2,3,17,31,32,48,52^. 

 Several studies have been conducted to improve understanding of the etiology of NEC and identify ways to prevent its progression, but few have yielded significant results that have led to changes in clinical practice. Some NBs present the disease in such a severe and acute form that morbidity and mortality are inevitable, even with treatment. Early detection of clinical and imaging signs can enable more accurate diagnosis and interventions^3,10,28,34,48^. 

 Genetic tests have been widely used in the investigation of several diseases, standing out for their minimally invasive nature and high sensitivity and specificity in molecular analysis, as demonstrated in the reference study^49^ and corroborated by the present study. 

 In the reference study^49^, the polymorphic G allele was identified in 4.9 and 3.9% of very low birth weight PTNBs with and without NEC, respectively, and in 6.5% of healthy FTNBs, with no significant differences between the groups. This indicates that there was no relationship between the presence of the polymorphism and the development of NEC in the analyzed case series^49^. However, this result was different from the present study, since, in the molecular analysis of the 200 alleles of the total case series, the polymorphic allele G was not identified in 100% of the cases. 

 To evaluate results in genetic association studies, it is essential that the groups analyzed share the same skin color and geographic region, since the genetic basis of several diseases, including the polymorphic configuration, can vary between populations and regions^56^. This aspect was considered in the present study, which, despite the Brazilian miscegenation, included patients and controls with apparent similarity in skin color and from the same geographic region. 

 The difference in molecular results in relation to the reference study^49^ may be related to the fact that it included only extremely low birth weight PTNBs, without mentioning ethnicity or skin color, in addition to the inclusion of NBs with other concomitant conditions and risk factors. In the present study, the inclusion and exclusion criteria were strictly followed to minimize bias in the results. 

 Although the results obtained by the molecular approach performed in the present study did not identify the 896A/G polymorphism in the *TLR4* gene, they should be interpreted with caution. Several genes and genetic interactions are involved in the development of NEC, the *TLR4* gene being just one of them^1,5,13,14,18,23,35,45,50^. 

 To better understand the complex genetic architecture of this condition, it is essential to continue independent and/or multicenter studies, aiming to determine the real prevalence of the 896A/G polymorphism in the *TLR4* gene and its association with NEC, both in international and Brazilian centers. 

 Understanding this molecular relationship is essential not only to deepen our knowledge of the physiology and pathophysiology of NEC, but also to identify its genetic causes. This could open up new perspectives for the development of more humanized and individualized protocols, including therapeutic schemes and technologies that enable early diagnosis and interventions, even in the face of these genetic alterations. 

## CONCLUSIONS

 The absence of the 896A/G polymorphism in the *TLR4* gene in NBs with and without NEC does not exclude the possibility of alterations in this and/or other genes in NBs affected by the disease, highlighting the importance of additional studies to elucidate this relationship. 

## Data Availability

The datasets generated and/or analyzed during the current study are available from the corresponding author upon reasonable request. The information regarding the investigation, methodology and data analysis of the article is archived under the responsibility of the authors.
